# Cross-Sectional Area Reference Values for Sonography of Peripheral Nerves in Taiwanese Adults

**DOI:** 10.3389/fneur.2021.722403

**Published:** 2021-11-03

**Authors:** Pei-Chen Hsieh, Kuo-Hsuan Chang, Yih-Ru Wu, Long-Sun Ro, Chun-Che Chu, Rong-Kuo Lyu, Ming-Feng Liao, Hung-Chou Kuo

**Affiliations:** ^1^Department of Neurology, Chang Gung Memorial Hospital at Linkou Medical Center, Taoyuan, Taiwan; ^2^Chang Gung University, College of Medicine, Taoyuan, Taiwan

**Keywords:** cross-sectional area, ultrasound, peripheral neuropathy, UPSS, Taiwan, sonography

## Abstract

**Background:** Neuromuscular ultrasound is a complementary technology that aids in the diagnosis of peripheral neuropathy. The interpretation of neuromuscular ultrasound results requires the use of accurate normative cross-sectional area (CSA) reference values. This study aims to provide CSA reference values specific to Taiwanese adults for Sonography of peripheral nerves in the upper and lower extremities.

**Methods:** The study cohort included 66 healthy subjects (36 women; 30 men). A linear probe was used to measure the CSA of the median, ulnar, radial, tibial, sural, and peroneal nerves at multiple sites. These data were analyzed to determine standard ranges for the CSA at each site (reference range = mean ± 2 × SD) and identify correlations between the CSA and patient characteristics.

**Results:** Normative CSA ranges were determined for all the assessed nerve sites, revealing that the nerve sizes in this Taiwanese population were smaller than Caucasian populations but comparable to those reported for other Asian cohorts. Men tended to have larger nerves than women, even after adjusting for height and weight. The size of ulnar nerve in the cubital tunnel and the peroneal nerve in the popliteal fossa correlated negatively with increasing age. The nerve size correlated positively with increasing weight and BMI at several sites, correlation of median nerve in the forearm with weight and BMI was significant after multiple testing. Significant correlation was also found between size of ulnar nerve in cubital tunnel and decreasing height.

**Conclusion:** We provide reference ranges for neuromuscular ultrasound CSA values for the upper and lower extremities that are specific to the Taiwanese population. These reference values may be useful for evaluating peripheral neuropathy in Taiwanese subjects.

## Introduction

Neuromuscular ultrasound (NMUS) is a point-of-care assessment that is increasingly used to acquire important morphological information for distinguishing peripheral nerve pathologies (1). NMUS provides complimentary information to electrodiagnostic studies and may assist clinicians in arriving at an accurate diagnosis of neuropathy. Additionally, NMUS improves the diagnostic yield in mononeuropathies by facilitating localization and aiding in the determination of the nature of the neuropathy (e.g., compression neuropathy vs. ganglion/cyst, tumor, or trauma) and in polyneuropathies (2, 3) by aiding in the identification of inflammatory neuropathies and distinguishing between different hereditary neuropathies (4–6). NMUS is also used for the diagnosis and treatment of entrapment and traumatic neuropathies (7–9).

The nerve cross-sectional area (CSA) is one of the most studied parameters for peripheral nerve evaluation that appears to be robust. The ultrasonic assessment of nerve enlargement is based on comparison to normative CSA values. However, the CSA reference values reported for peripheral nerves vary between studies. Previous studies have established that normal CSA values for specific nerves vary according to ethnicity (10), age (11–14), and sex (15). Further, the reported CSA reference values vary even within the Asian population (14, 16–18). Thus, the differences in reported normative CSA values may be due in part to differences in the study cohort characteristics. These findings suggest that accurate interpretation of nerve sonography data requires the use of CSA reference values that are specific to each ethnic group.

Normative CSA reference values for the Taiwanese population have not yet been established. This study aims to determine the CSA reference value ranges for sonography of peripheral nerves in the upper and lower extremities of Taiwanese adults.

In a cohort of healthy Taiwanese participants, we conducted NMUS at multiple nerve sites in the upper and lower extremities to determine the baseline CSAs.

## Methods

### Patients and Their Clinical Data

The study included 66 healthy participants aged 20–75 years recruited at a single Medical Center from January 2020 to May 2021. The participants included healthy hospital volunteers, hospital staff, and outpatients without neurological, neuropathy, neuromuscular medical conditions or disorders (thereby excluding patients with previous diagnosis, clinical signs of neuropathy). All participants underwent neurological examination and/or electrophysiology studies, and their medical histories were recorded. Exclusion criteria were as follows: diagnosis of focal neuropathy or polyneuropathy disorders; systemic diseases including liver cirrhosis, chronic renal disease, malignancy, diabetes mellitus, thyroid disease, and autoimmune disease associated with vasculitis; recent history of pregnancy; exposure to a neurotoxic agent or heavy metals; family history of hereditary peripheral neuropathy; and abuse of illicit drugs or alcoholism within 1 year, as these conditions may lead to neuropathy disorders.

None of the subjects had abnormal sensory or motor signs, as indicated by a neurological examination assessing sensory function, muscle strength, and deep tendon reflexes. Conduction studies were administered to 45 individuals by three experienced technicians with more than 20 years of experience performing electrophysiology studies. Standard sensory and motor nerve conduction studies results were normal in the median, ulnar, tibial, peroneal, and sural nerves in all participants. The study was approved by the hospital Institutional Review Board (IRB202100356B0) and informed consent was obtained from all subjects.

### Peripheral Nerve Ultrasound

The electrodiagnostic testing protocol included assessment of the compound muscle action potential, sensory action potential, and conduction velocities, carried out as described previously (19, 20). Nerve sites measured included the median, ulnar, tibial, peroneal, and sural nerves. Nerve segments sampled were: (1) Median motor conduction study (NCS): stimulation of wrist and antecubital fossa, measurements recorded from the abductor pollicis brevis. (2) Ulnar motor study: stimulation of wrist, below groove, and at a distance of 10–12cm from the below-elbow site over middle humerus in flexed position, measurements recorded from abductor digiti minimi. (3) Median sensory conduction study: stimulation of mid-wrist between the tendons to the flexor carpi radialis and palmaris longus, measurements recorded from digit 2^nd^ or 3^rd^. (4) Ulnar sensory response: stimulation of wrist, measurements recorded from digit 5^th^. (5) Peroneal motor study: stimulation of the ankle, below fibular head, and at a 10–12 cm from the below–fibular head site near external hamstring tendon, measurements recorded from the extensor digitorum brevis. (6) Tibial motor study: stimulation of medial ankle and popliteal fossa, measurements recorded from abductor hallucis brevis. (7) Sural sensory study: stimulation of segment 14 cm from lateral malleolus in calf, measurements recorded from the posterior ankle. The normal reference of all NCS parameters was according to our previous study (19). The stimulation was under distal limb temperature around 32-34°C.

All subjects were assessed using a multifrequency linear transducer 4-15Hz (UP200, BenQ Medical Technology, Corp., Taipei, Taiwan) in B mode. During the examination, the ultrasound device frequency automatically adjusted to the higher frequency, and the gain and dynamic scan were kept constant. The focus and depth were set depending on the distance from the skin to the target point. Zooming in was avoided to maintain consistency in measurements. The ultrasound examinations were unilateral, and were conducted by one neurologist with 2 year neuromuscular ultrasound experience.

**Figure 1:**
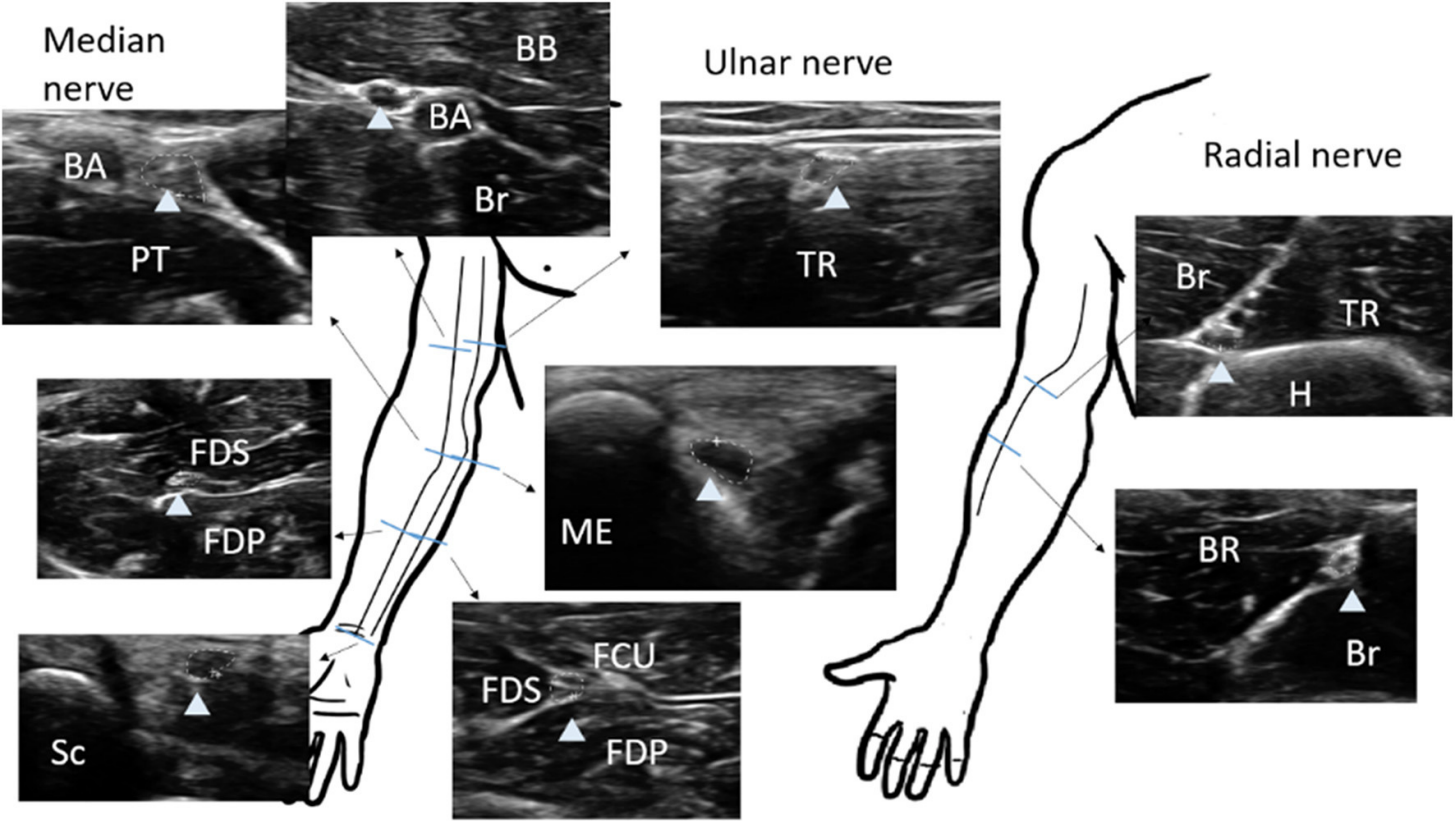
Sites of nerve ultrasonography to determine the cross-sectional area (CSA) in the median, ulnar, and radial nerves. The CSA of each nerve (arrow head) is measured within the hyperechoic rim (dotted line). Median nerve sites: wrist, mid-forearm, antecubital fossa, and mid-arm. Ulnar nerve sites: mid-forearm, cubital tunnel, and mid-arm. BA, brachial artery; BB, biceps brachii; Br, brachialis; FCU, flexor carpi ulnaris; FDS, flexor digitorum superficialis; FDP, flexor digitorum profunuds; ME, medial epicondyle; SB, scaphoid bone; PT, pronator teres; TR, triceps.

**Figure 2:**
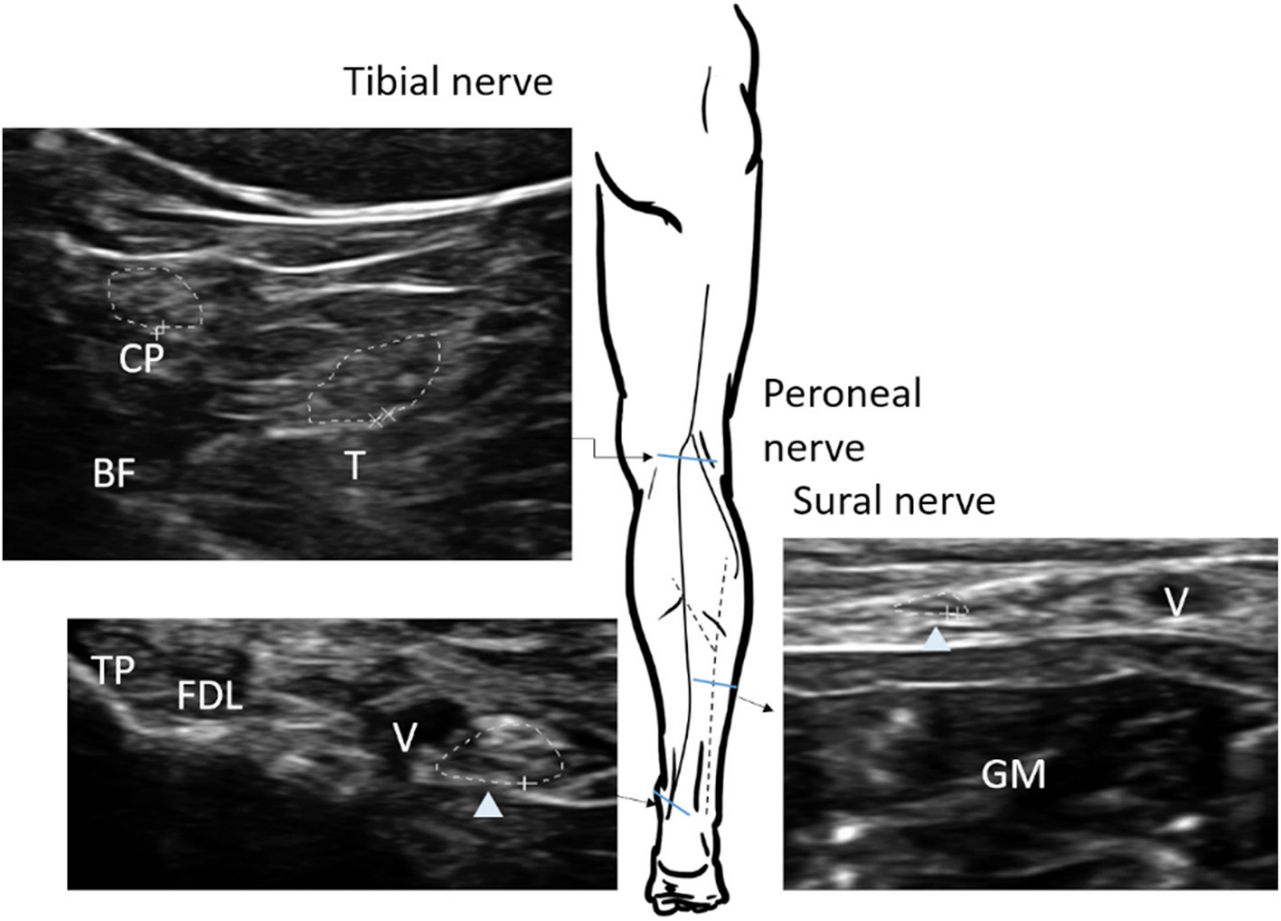
Sites of nerve ultrasonography to determine the cross-sectional area (CSA) in the tibial, peroneal, and sural nerves. The CSA of each nerve (arrow head) is measured within the hyperechoic rim (dotted line). The tibial and peroneal nerves in popliteal fossa are defined as the location of the split by the sciatic nerve; sural nerve, 10 cm proximal to the lateral malleolus of the calf next to the small saphenous vein. BF, biceps femoris muscle; T, tibial nerve; CP, common peroneal nerve; GM, gastrocnemius; TP, tibialis posterior; FDL, flexor digitorum longus; V, vein.

The nerve CSA was determined by tracing the nerve area within the hyperechoic epineurium. Each selected nerve was measured two times (tibial and peroneal nerves were tested at least three times due to the relatively indistinct border) on separate days with a minimal of 2-day interval in between each measurement, and raters were blinded to previous results. The intra-rater reliability, and inter-rater reproducibility was >0.85 for all ultrasound measurements (Supplementary Table 1). The CSA was measured in the median nerve at the mid-upper arm, the cubital area next to brachial artery, the mid-forearm, and the wrist; the ulnar nerve was measured at the mid-upper arm and the mid-forearm (Figure 1). The common peroneal and tibial nerves were measured at the popliteal fossa, the tibial nerve was measured at the ankle, and the sural nerve was measured 10 cm proximal to the lateral malleolus next to the small saphenous vein (Figure 2).

We then used the CSA values to determine the ultrasound pattern sum score (UPSS), defined as the CSA measurement at the median nerve non-entrapment area (mid-forearm, elbow, mid-upper arm), ulnar nerve (mid-forearm and mid-upper arm), tibial nerve (popliteal fossa and ankle), peroneal nerve at the popliteal fossa, and sural nerve at the calf. Each value > 100% of twice the standard deviation (SD) was scored as one point; scores >150% received two points (2).

### Statistical Analysis

Demographic data are reported as the mean ± standard deviation (SD) and range. The Mann–Whitney *U*-test was used to evaluate differences in continuous epidemiological data (age, height, weight, and body mass index [BMI]). CSA measurements are reported as the mean ± SD by group. The CSA reference range is reported as the mean ± 2 SD for all subjects and in subgroups of males and females. Differences in CSA measurements between the sexes groups were evaluated using the multivariate linear regression analysis adjusting for age, height, and weight. Multivariate linear regression analysis was also performed to test for linear correlations between nerve size and weight, height, and BMI with adjustments for sex and age. All results are presented as the coefficient of regression (ß) with the corresponding *P* value. Multiple testing correction was performed using false discovery rate method with q^*^ = 0.05. Statistical analysis was conducted using SPSS version 22.0 (IBM Corporation, Armonk, NY, USA). *P* < 0.05 was considered statistically significant.

## Results

### Demographic Data

The mean age of the 66 enrolled subjects (30 men and 36 women) was 42.1 ± 14.0 (range, 20–74). No difference was found in age, or BMI, between male and female sex. However, men were taller and heavier than the women (*P* < 0.01). The participant characteristics are summarized in Table 1.

**Table 1:** Cohort characteristics.

	**All**	**Male**	**Female**	***P***
Number	66	30	36	
Age range, years	20–74	21–74	20–74	
Age, years^#^	42.1 ± 14.0	42.0 ± 14.3	42.1 ± 14.0	0.959
Height, cm ^#^	163.2 ± 8.1	168.8 ± 8.4	158.3 ± 5.9	<0.01^**^
Weight, kg ^#^	63.6 ± 11.3	69.9 ± 10.5	58.4 ± 9.2	<0.01^**^
Body mass index, kg/m^2#^	23.9 ± 3.3	24.5 ± 3.1	23.4 ± 3.4	0.153

** *Statistically significant difference; P < 0.01*.

# *Mean ± standard deviation*.

### Mean CSA and Its Correlation With Demographic Factors

**Figure 3:**
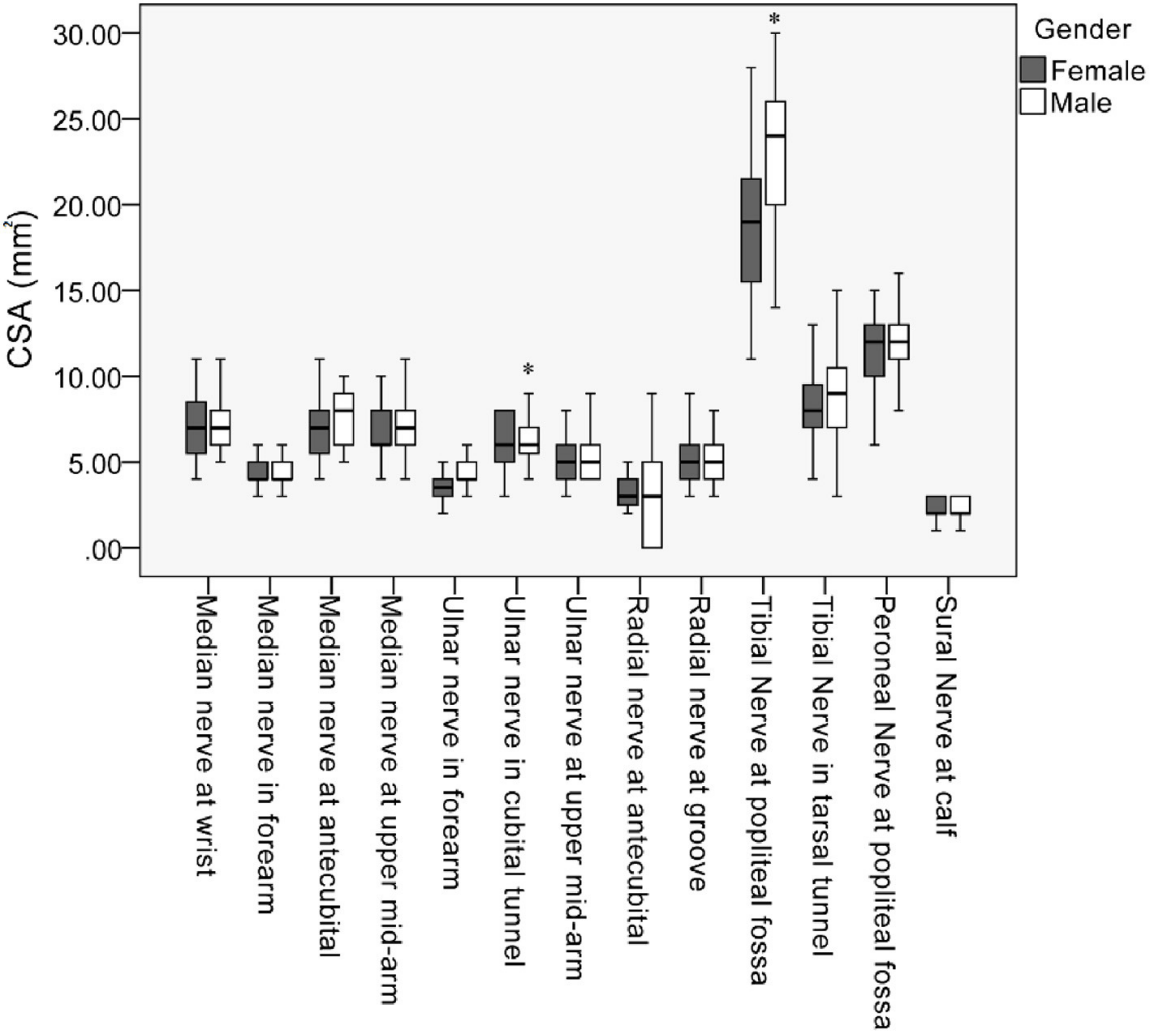
Box plot of cross-sectional area (CSA) values along the median, ulnar, radial, peroneal, and sural nerves. ^*^Statistically significant differences of CSA between male and female in the mean ulnar nerve in cubital tunnel and tibial nerve at popliteal fossa (*P* < 0.05).

**Figure 4:**
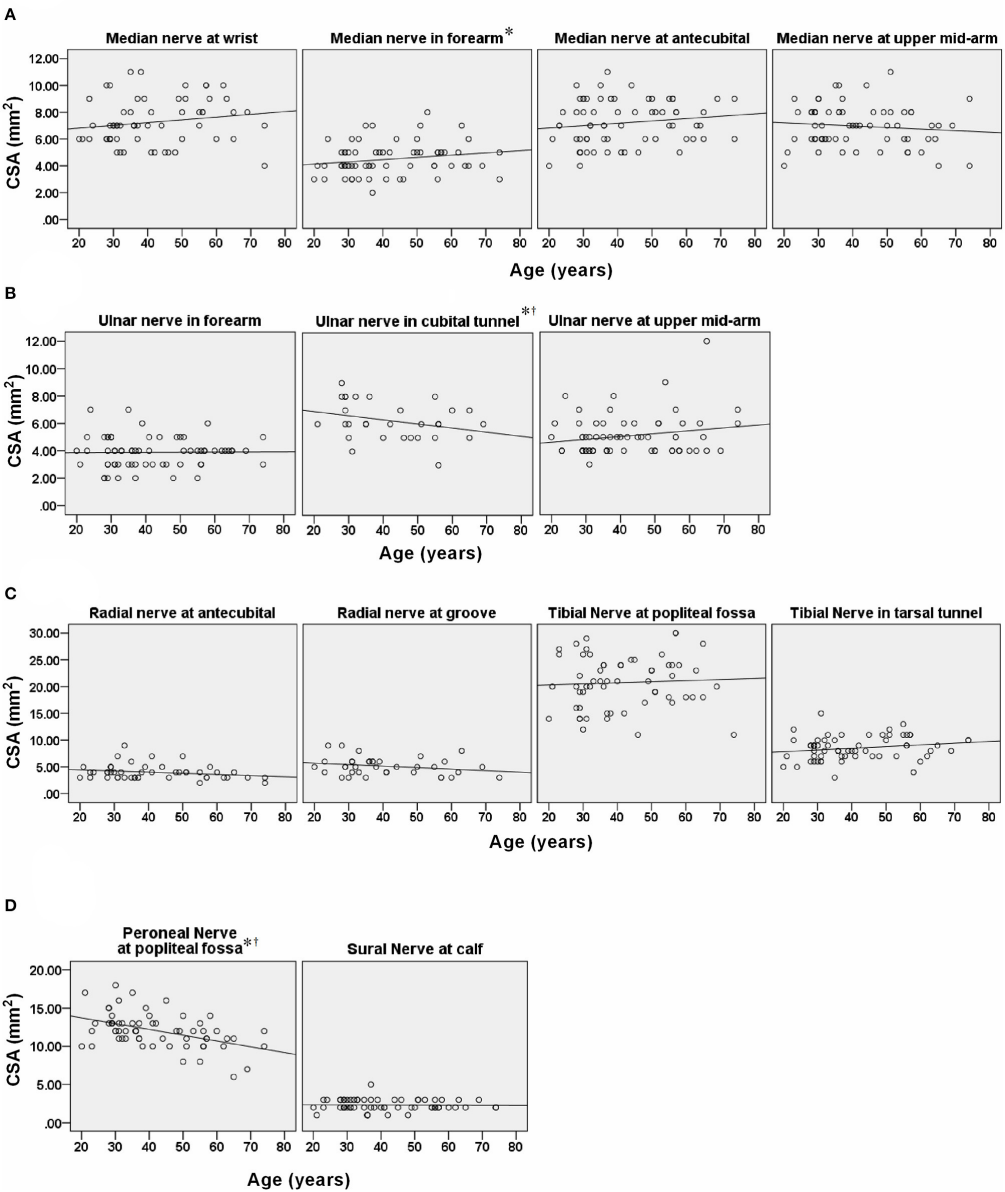
Scatter plot showing correlation of age with cross-sectional area (CSA) at different sites of nerve. **(A)** median nerves. **(B)** ulnar nerves. **(C)** radial nerves and **(D)** peroneal and sural nerves. ^*^Statistically significant correlation in: median nerve in the forearm, ulnar nerve in cubital tunnel, and peroneal nerve in the popliteal fossa. Statistically significant correlation under false discovery rate multiple testing method.

The mean values, SD, and reference range of all measurements of all subjects are summarized in Table 2. The nerves in the lower extremities were larger than those in the upper extremities. In most nerves, men tended to have a larger CSA than did the women. Common sites of entrapment had a larger CSA. Differences in the CSA were observed between the sexes in the ulnar nerve (cubital tunnel, *P* = 0.035) and tibial nerve (popliteal fossa, *P* = 0.014) (Table 2; Figure 3). Further analysis by age showed a larger CSA in the median nerve in the forearm (*P* = 0.016) with increasing age. By contrast, the decreasing CSA in ulnar nerve (cubital tunnel, *P* = 0.002) and peroneal nerves at the popliteal fossa was associated with increasing age (*P* = 0.005) (Table 3). The association of decreasing CSA in ulnar nerve (cubital tunnel) and peroneal nerves at the popliteal fossa with increasing age remain statistically significant after correction using FDR. The scatter plot of participants' age vs. CSA size at different sites is shown in Figure 4.

**Table 2:** Peripheral nerve cross-sectional areas with reference ranges according to sex.

	**All**		**Male**		**Female**		
	**Mean ± SD**	**Reference range^***a***^**	**Mean ± SD**	**Reference range^***a***^**	**Mean ± SD**	**Reference range^***a***^**	***P***
**Median nerve**							
Median nerve at wrist	6.8 ± 2.4	2.0–11.5	7.0 ± 2.4	2.0–11.5	6.7 ± 2.4	2.0–11.5	0.683
Median nerve in forearm	4.5 ± 1.2	2.0–7.0	4.6 ± 1.2	2.0–7.0	4.4 ± 1.2	2.0–7.0	0.064
Median nerve at antecubital	7.2 ± 1.6	4.0–10.5	7.7 ± 1.6	4.5–10.5	6.8 ± 1.6	3.5–10.5	0.605
Median nerve at upper mid-arm	7.0 ± 1.5	4.0–10.0	7.2 ± 1.6	4.0–10.5	6.7 ± 1.5	4.0–9.5	0.487
**Ulnar nerve**							
Ulnar nerve in forearm	3.9 ± 1.1	2.0–6.0	4.2 ± 1.1	2.0–6.0	3.6 ± 1.2	1.0–6.0	0.588
Ulnar nerve in cubital tunnel	6.2 ± 1.4	3.0–9.0	6.3 ± 1.3	3.5–9.0	6.2 ± 1.4	3.0–9.0	0.035^*^
Ulnar nerve at upper mid-arm	5.1 ± 1.5	2.0–8.0	5.2 ± 1.8	2.0–8.0	5.0 ± 1.2	2.5–7.0	0.303
**Radial nerve**							
Radial nerve at antecubital	4.0 ± 1.4	1.0–7.0	4.6 ± 1.8	2.0–7.5	3.6 ± 0.7	2.0–5.0	0.945
Radial nerve at groove	5.1 ± 1.6	2.0–8.0	4.8 ± 1.5	2.0–8.0	5.4 ± 1.6	2.0–8.0	0.756
**Tibial nerve**							
Tibial Nerve at popliteal fossa	20.8 ± 4.8	11–30	23.2 ± 4.5	14.5–32	18.9 ± 4.3	10–27.5	0.014^*^
Tibial Nerve in tarsal tunnel	8.5 ± 2.2	4.0–13	8.8 ± 2.4	4.0–13.5	8.3 ± 2.1	2–12.5	0.429
**Peroneal nerve**							
Peroneal nerve at popliteal fossa	12.1 ± 2.2	7.5–16	12.1 ± 2.5	7.0–17	12 ± 2.0	2.0–16	0.221
**Sural nerve**							
Sural nerve at calf	2.3 ± 0.7	0.8–3.5	2.4 ± 0.5	1.0–3.5	2.3 ± 0.8	0.5–3.5	0.450

* *Statistically significant difference; P < 0.05*.

*SD, standard deviation*.

a *Reference range = mean ± (2 × SD)*.

**Table 3:** Peripheral nerve cross-sectional area reference values according to age.

	**ß**	***P*-value**
**Median nerve**		
Median nerve at wrist	0.111	0.461
Median nerve in forearm	0.335	0.016^*^
Median nerve at antecubital	0.219	0.139
Median nerve at upper mid-arm	−0.140	0.361
**Ulnar nerve**		
Ulnar nerve in forearm	0.138	0.357
Ulnar nerve in cubital tunnel	−0.619	0.002^*^^†^
Ulnar nerve at upper mid-arm	0.300	0.052
**Radial nerve**		
Radial nerve at antecubital	−0.230	0.120
Radial nerve at groove	−0.232	0.302
**Tibial nerve**		
Tibial Nerve at popliteal fossa	0.071	0.620
Tibial Nerve in tarsal tunnel	0.145	0.321
**Peroneal nerve**		
Peroneal Nerve at popliteal fossa	−0.398	0.005^*^^†^
**Sural nerve**		
Sural Nerve at calf	0.036	0.818

* *Statistically significant difference; P < 0.05*.

† *Statistically significant difference with false discovery rate controlling method*.

A positive correlation was observed between the CSA of several nerve sites with body weight: the median nerve in the forearm (*P* = 0.001) and upper arm (*P* = 0.047); the ulnar nerve in the cubital tunnel (*P* = 0.035); the radial nerve in the groove (*P* = 0.044) and the antecubital (*P* = 0.019); and the tibial nerve in the tarsal tunnel (*P* = 0.022). In contrast, only the ulnar nerve in the cubital (*P* < 0.001) correlated with height. The median nerve at the wrist (*P* = 0.043) and upper mid-arm (*P* = 0.043), radial nerve at the antecubital (*P* = 0.032), and the tibial nerve in the tarsal tunnel (*P* = 0.017) correlated with BMI (Table 4). The correlation remained significant after correction using FDR between CSA of median nerve in the forearm with body weight and BMI, and CSA of ulnar nerve in the cubital tunnel and height (Table 4).

**Table 4:** Multivariant analysis of CSA values with respect to height and weight.

	**Weight**		**Height**		**BMI**	
**Site of CSA measurement**	**ß**	***P***	**ß**	**P**	**ß**	***P***
**Median nerve**						
Median nerve at wrist	0.332	0.053	0.044	0.840	0.271	0.043^*^
Median nerve in forearm	0.531	0.001^*^^†^	0.059	0.765	0.429	0.001^*^^†^
Median nerve at antecubital	0.128	0.548	0.186	0.262	0.146	0.263
Median nerve at upper mid-arm	0.344	0.047*	−0.213	0.337	0.270	0.043*
**Ulnar nerve**						
Ulnar nerve in forearm	0.164	0.331	0.118	0.586	0.116	0.380
Ulnar nerve in cubital tunnel	0.392	0.035*	−1.04	<0.001^*^^†^	0.365	0.051
Ulnar nerve at upper mid-arm	0.194	0.259	0.196	0.374	0.144	0.288
**Radial nerve**						
Radial nerve at antecubital	0.396	0.019*	−0.332	0.122	0.283	0.032*
Radial nerve at groove	0.484	0.044*	−0.493	0.145	0.360	0.050*
**Tibial nerve**						
Tibial nerve at popliteal fossa	0.257	0.107	−0.232	0.263	0.211	0.098
Tibial nerve in tarsal tunnel	0.392	0.022*	−0.228	0.310	0.317	0.017*
**Peroneal nerve**						
Peroneal nerve at popliteal fossa	−0.002	0.989	0.246	0.228	−0.010	0.936
**Sural nerve**						
Sural nerve at calf	0.275	0.127	0.035	0.886	0.214	0.128

*BMI, body mass index*.

* *Statistically significant difference; P < 0.05*.

† *Statistically significant difference with false discovery rate controlling method*.

### Adjusted Ultrasound Pattern Sum Score

Using our CSA reference values, we adjusted the UPSS score make it more suitable for use in Taiwanese subjects (Supplementary Table 2). Applying this score to our healthy controls revealed that a UPSS score < 3 was defined as normal, and 85% of our participants had a UPSS = 0. All participants had a UPSS < 3.

## Discussion

In a cohort of healthy Taiwanese adults, we measured the CSA of extremity nerves at multiple sites to determine normal CSA reference values for this population. The CSA values observed in our study cohort were smaller than those reported in studies of other Asian and Caucasian cohorts, supporting our proposal that the ethnic differences in nerve size necessitates the establishment of population-specific CSA reference values. In addition, after correction for multiple testing, we observed that age correlated negatively with the size of the ulnar nerve in the cubital tunnel and the peroneal nerve in the popliteal fossa. Weight, and body mass index were associated with nerve size at several sites. Men had larger CSAs in the upper limbs and proximal tibial nerve, after statistical adjustment for weight and height. These findings should be useful for the analysis of nerve sonography data specific to the Taiwanese population.

The relative sizes of the nerves observed in this study were similar to those reported in other cohorts. In the upper extremities, the median nerve CSA tended to be larger than either the ulnar nerve or the radial nerve in most of the segments. In the forearm, the CSAs of the median and ulnar nerves were smaller than those of the other segments. The CSAs of nerves located in the lower extremities were larger than those of the upper extremities. Several studies report similar findings, with relatively large CSAs in the common entrapment sites (10, 21).

As observed in our cohort, several previous studies report correlation between CSA values and sex, with men having slightly larger nerves than women (2, 15, 16, 21). In our cohort, men had larger CSAs in the upper limbs and proximal tibial nerve than did women, even after statistical adjustment for weight and height; the other targeted nerve sites exhibited this tendency, but without statistical significance.

Many studies investigating the factors that influence the CSA report a generally positive correlation between nerve size and age (11–16, 21). However, Kerasnoudis et al., reported a paradoxical result of decreasing nerve size with increasing age in the median nerve in the axilla and the radial nerve in the spiral groove (22). Our results showed a significant increase with age in the CSA of the median nerve of the forearm. However, in the ulnar nerve in cubital the tunnel and the peroneal nerve in the popliteal fossa, we observed a decrease in CSA with age.

Studies on the correlation between CSA and weight, height, and BMI have reported varied results. Soek et al. and Cartwright et al., found that the CSA correlated with height, body weight, and BMI in the peroneal nerve and the tibial nerve (11, 18); In contrast, other studies found no correlations with the CSA (21, 22). Won et al., showed a correlation between these parameters in most segments of the median, ulnar, and radial nerves (16). This is similar to our finding, where correlations with CSA were found in the median nerve in the forearm (weight) and upper mid-arm (weight and BMI), the ulnar nerve in the cubital (weight and height), the radial nerve in the antecubital (weight and BMI) and groove (weight and BMI), and the tibial nerve in the tarsal tunnel (weight and BMI).

In addition to age, weight, height, and sex, evidence suggests that CSA values are influenced by ethnicity, which may contribute to the differences in reported CSA values between study cohorts (10). Comparison of the CSA values of our Taiwanese cohort to those of other studies (Table 5) revealed that our cohort CSAs are smaller than those reported in studies of Caucasian cohorts, a finding supported by numerous previous studies comparing Asian and Caucasian nerve sizes (10, 15, 21). In cohorts from Germany, the USA, and Canada, the targeted upper limb nerves had a larger CSA than in our cohort. The USA cohort had a larger CSA for the tibial nerve in the popliteal area and sural nerve than did other cohorts. Asians tend to be of lower weight and height than Caucasians, which may contribute to the difference in nerve size observed between these populations. As shown in Table 4, we observed a positive correlation between weight and nerve size in our cohort. This finding is supported by a recent study (25) in which weight was found to be the body habitus parameter that most influenced the nerve CSA; in contrast, height did not predict the CSA magnitude. The upper extremity nerve CSAs are larger in Koreans than in other Asians, more similar in size to those observed in Caucasians (16, 18). While the CSA of the sural nerve does not differ between Asians and Caucasians, differences have been observed between these populations in the CSAs of the tibial and peroneal nerves in the popliteal fossa. The abundant connective tissue around these nerves can render the nerve boundary difficult to distinguish, possibly contributing to these conflicting findings. The CSA values of our cohort were also smaller than those in other Asian cohorts (Table 5). These findings suggest that for accurate analysis of nerve sonography results, normal CSA reference values should be established for specific ethnic groups.

**Table 5:** Comparison of published CSA values from cohorts of different ethnicities.

	**Taiwan**	**Germany**	**Germany**	**USA**	**Canada**	**China**	**Korea**	**Japan**	**India**
	**Our study**	**Grimm et al**. **(****15****)**	**Kerasanoudis et al**. **(****22****)**	**Cartwright et al**. **(****11****)**	**Qrimli et al**. **(****23****)**	**Niu et al**. **(****21****)**	**Won et al**. **(****16****)/ Soek et al**. **(****18****)**	**Sugimoto et al**. **(****14****)**	**Bathala et al**. **(****24****)**
**Median nerve**									
Median nerve at wrist	6.8 ± 2.4	10.6 ± 2.9	8.4 ± 2.1	N/A	10.0 ± 2.4	6.3 ± 0.9	9.3 ± 1.6	8.5 ± 1.7	7.2 ± 1
Median nerve in mid-forearm	4.5 ± 1.2	7.2 ± 1.3	6.6 ± 1.6	N/A	7.3 ± 1.7	5.6± 0.9	6.3 ± 1.5	6.0 ± 1.3	4.8 ± 0.9
Median nerve at antecubital	7.2 ± 1.6	9.2 ± 1.7	N/A	N/A	10.3 ± 3.4	8.4± 1.3	9.0 ± 2.4	9.1 ± 2.2	N/A
Median nerve at mid-arm	7.0 ± 1.5	9.1 ± 1.5	N/A	N/A	9.4 ± 3.1	7.8± 1.2	9.3 ± 2.5	5.6 ± 1.0	6.1 ± 1
**Ulnar nerve**									
Ulnar nerve in mid-forearm	3.9 ± 1.1	5.9 ± 1.4	N/A	N/A	6.2 ± 1.5	4.6± 0.8	N/A	4.7 ± 1.0	N/A
Ulnar nerve in cubital tunnel	6.2 ± 1.4	8.7 ± 2.0	5.5 ± 1.3	N/A	6.9 ± 2.3	5.6± 1.1	7.3 ± 1.7	6.7 ± 1.9	N/A
Ulnar nerve at mid-arm	5.1 ± 1.5	7.0 ± 1.2	N/A	N/A	6.8 ± 2.3	4.4± 0.9	6.4 ± 1.6	4.8 ± 1.0	N/A
**Radial nerve**									
Radial nerve at antecubital	4.0 ± 1.4	N/A	N/A	9.3± 2.4	N/A	N/A	7.3 ± 1.7	N/A	N/A
Radial nerve at groove	5.1 ± 1.6	N/A	3.3 ± 1.5	N/A	6.5 ± 1.7	N/A	6.8 ± 1.8	N/A	N/A
**Tibial nerve**									
Tibial nerve at popliteal fossa	20.8 ± 4.8	23.2 ± 4.9	N/A	35.3 ± 10.3	N/A	N/A	24.4± 4.4	N/A	N/A
Tibial nerve in tarsal tunnel	8.5 ± 2.2	10.2 ± 2.0	6.4 ± 1.5	13.7 ± 7.3	12.7 ± 3.4	N/A	N/A	N/A	N/A
**Peroneal nerve**									
Peroneal nerve at popliteal fossa	12.1 ± 2.2	8.4 ± 1.6	8.6 ± 1.8	11.7 ± 4.6	11.8 ± 3.8	N/A	10.4 ± 2.7	N/A	N/A
**Sural nerve**									
Sural nerve at calf	2.3 ± 0.7	2.2 ± 0.6	N/A	5.3 ± 1.8	2.1 ± 0.8	N/A	2.6 ± 0.6	N/A	N/A

*Data are presented as the mean ± SD*.

*N/A, not available*.

The CSA data for peripheral nerves at multiple sites can be evaluated collectively using a variety of methods, including the UPSS. As a starting point for testing the use of our new CSA standard values for Taiwanese, we used the UPSS scoring system because UPSS is able to quantify nerve enlargement at several nerve segments (Supplementary Table 2). Applying this score to our healthy cohort showed that a UPSS score < 3 is defined as normal. All of our participants had UPSS < 3, and 85% had UPSS = 0, and these values are similar to those reported in a German cohort (2, 15). These findings suggest that slight variations exist within the normal population. Since UPSS scores have not yet been validated using independent samples, further studies assessing several additional nerve sites is warranted to refine the total score. In addition, various ultrasound scoring tools are available and have been shown applicable with high accuracy (1, 26), tools in addition to UPSS should be considered in the future.

This preliminary study has several limitations. First, the study cohort was relatively small. However, other studies of similar cohort size using unilateral measurements have been published (15, 27–29). Second, the extremity nerve measurements were only unilateral. However, previous studies have shown that there were only minimum detectable differences or no statistically significant side-to-side differences in CSA (30, 31). Third, the cut-off values for the reference range and the UPSS are not adjusted in all epidemiological subgroups. We used double the SD as the normal reference range to exclude possible influencing factors. We measured the CSA in the extremities but not in the cervical root. Since the diagnosis of some peripheral neuropathies requires root values, future studies should be conducted to establish the normal reference values for the cervical root, in addition to other common entrapment sites including ulnar CSA at the wrist, and fibular nerve at the fibular head.

## Conclusion

Different populations may have distinct CSA values according to racial or ethnic variations. This study establishes a CSA reference range specific to the Taiwanese population for use in nerve sonography. This work may be helpful for the evaluation of peripheral nerve disorders in specific populations.

## Data Availability Statement

The raw data supporting the conclusions of this article will be made available by the authors, without undue reservation.

## Ethics Statement

The studies involving human participants were reviewed and approved by institutional review board of Chang Gung Memorial Hospital (IRB202100356B0 and IRB 202101058B0). The patients/participants provided their written informed consent to participate in this study.

## Author Contributions

All authors provided healthy participants. H-CK conceived, designed the study, and performed the experiments. P-CH performed the ultrasound in all participants. P-CH and H-CK analyzed the data and then wrote the paper. P-CH, H-CK, K-HC, Y-RW, L-SR, C-CC, R-KL, M-FL read and approved the final manuscript.

## Funding

This research was supported in part by Chang Gung Memorial Hospital (CMRPG3L1121, CMRPG3H1761 and CMRPG3J1751).

## Conflict of Interest

The authors declare that the research was conducted in the absence of any commercial or financial relationships that could be construed as a potential conflict of interest.

## Publisher's Note

All claims expressed in this article are solely those of the authors and do not necessarily represent those of their affiliated organizations, or those of the publisher, the editors and the reviewers. Any product that may be evaluated in this article, or claim that may be made by its manufacturer, is not guaranteed or endorsed by the publisher.

## Abbreviations

BMI: body mass index
CIDP: chronic inflammatory demyelinating polyradiculopathy
CSA: cross-sectional area
ICC: intraclass correlation coefficient
NMUS: neuromuscular ultrasound
SD: standard deviation
UPSS: ultrasound pattern sum score.
