# Supplementary material for: Cross-Sectional Area Reference Values for Sonography of Peripheral Nerves in Taiwanese Adults
Source: Front Neurol. 2021 Nov 3;12:722403. doi: 10.3389/fneur.2021.722403 (PMC8596614; doi:10.3389/fneur.2021.722403)
Supplement: Supplementary file 1 [file Data_Sheet_1.docx]

Supplemental Table 1. Intra-rater and inter-rater correlations

|  |  | Intra-rater correlation 1 | Intra-rater correlation 2 | Inter-rater correlation |
| --- | --- | --- | --- | --- |
|  |  |  |  |  |
| **Median Nerve** |  |  |  |  |
| Median nerve at wrist |  | 0.965 | 0.934 | 0.927 |
| Median nerve at cubital |  | 0.979 | 0.921 | 0.819 |
| Median nerve at upper mid-arm |  | 0.994 | 0.923 | 0.837 |
| **Ulnar Nerve** |  |  |  |  |
| Ulnar nerve at upper mid-arm |  | 0.931 | 0.897 | 0.851 |
| **Tibial Nerve** |  |  |  |  |
| Tibial Nerve at popliteal fossa |  | 0.992 | 0.920 | 0.866 |
| Tibial Nerve in tarsal tunnel |  | 0.988 | 0.891 | 0.858 |
| **Peroneal Nerve** |  |  |  |  |
| Peroneal Nerve at popliteal fossa |  | 0.944 | 0.859 | 0.835 |

Supplemental Table 2. Study cohort ultrasound pattern sum scores (UPSS)

|  | | UPSS | cut-off (mean + 2 × SD |  |  |
| --- | --- | --- | --- | --- | --- |
| Peripheral nerve | | Boundary value (mm^2^) | Normal <50%/>50% | Points |  |
| Median nerve | |  |  |  |  |
|  | Upper arm | 10 | <10 | 0 |  |
|  |  |  | ≧10, ≦15 | 1 |  |
|  |  |  | >15 | 2 |  |
|  | Elbow | 10.5 | <10.5 | 0 |  |
|  |  |  | ≧10.5, ≦15.75 | 1 |  |
|  |  |  | >15.75 | 2 |  |
|  | Forearm | 7 | <7 | 0 |  |
|  |  |  | ≧7, ≦10.5 | 1 |  |
|  |  |  | >10.5 | 2 |  |
| Ulnar nerve | |  |  |  |  |
|  | Upper arm | 8 | <8 | 0 |  |
|  |  |  | ≧8, ≦12 | 1 |  |
|  |  |  | >12 | 2 |  |
|  | Forearm | 6 | <6 | 0 |  |
|  |  |  | ≧6, ≦9 | 1 |  |
|  |  |  | >9 | 2 |  |
| Tibial nerve | |  |  |  |  |
|  | Popliteal | 30 | <30 | 0 |  |
|  |  |  | ≧30, ≦45 | 1 |  |
|  |  |  | >45 | 2 |  |
|  | Ankle | 13 | <13 | 0 |  |
|  |  |  | ≧13, ≦19.5 | 1 |  |
|  |  |  | >19.5 | 2 |  |
| Peroneal nerve | |  |  |  |  |
|  | Popliteal | 16 | <16 | 0 |  |
|  |  |  | ≧16, ≦24 | 1 |  |
|  |  |  | >24 | 2 |  |
| Sural nerve | |  |  |  |  |
|  | Calf | 3.5 | <3.5 | 0 |  |
|  |  |  | ≧3.5 | 1 |  |
|  | Total score |  |  | 0-17 |  |

UPSS: ultrasound pattern sum score
